# Should applicants to Nottingham University Medical School study a non-science A-level? A cohort study

**DOI:** 10.1186/1472-6920-9-5

**Published:** 2009-01-21

**Authors:** Janet Yates, Jennifer Smith, David James, Eamonn Ferguson

**Affiliations:** 1 Medical Education Unit, B94 Medical School, Queen's Medical Centre, Nottingham, NG7 2UH, UK; 2 Health Psychology, University of Nottingham, University Park, Nottingham, NG7 2RD, UK

## Abstract

**Background:**

It has been suggested that studying non-science subjects at A-level should be compulsory for medical students. Our admissions criteria specify only Biology, Chemistry and one or more additional subjects. This study aimed to determine whether studying a non-science subject for A-level is an independent predictor of achievement on the undergraduate medical course.

**Methods:**

The subjects of this retrospective cohort study were 164 students from one entry-year group (October 2000), who progressed normally on the 5-year undergraduate medical course at Nottingham. Pre-admission academic and socio-demographic data and undergraduate course marks were obtained. T-test and hierarchical multiple linear regression analyses were undertaken to identify independent predictors of five course outcomes at different stages throughout the course.

**Results:**

There was no evidence that the choice of science or non-science as the third or fourth A-level subject had any influence on course performance. Demographic variables (age group, sex, and fee status) had some predictive value but ethnicity did not. Pre-clinical course performance was the strongest predictor in the clinical phases (pre-clinical Themes A&B (knowledge) predicted Clinical Knowledge, p < 0.001, and pre-clinical Themes C&D (skills) predicted Clinical Skills, p = < 0.01).

**Conclusion:**

This study of one year group at Nottingham Medical School provided no evidence that the admissions policy on A-level requirements should specify the choice of third or fourth subject.

## Background

In 2006, approximately 19,000 students made 75,000 applications for 8,000 medical school places in the UK.[1] Applications are made centrally via the Universities and Colleges Admissions Service (UCAS).[2] Candidates may apply for up to four separate medical schools each October, for admission in the following academic year. The application form requires candidates to list their current school examination results and their Head Teachers to provide predicted grades for final examinations. The majority of applicants to medical schools in the UK will be studying three or four subjects at General Certificate of Education Advanced level (GCE A-level) in their last year at school, although some follow alternative qualifications such as Scottish Highers or the International Baccalaureate. Although selection criteria vary between Institutions, and there are various moves to widen admission criteria.[3], the majority of 5-year undergraduate medical courses still place great emphasis on A-levels or equivalent.[4]

A-levels have been extensively studied. Good science grades are related to success on the undergraduate course [5-9], a lower drop-out rate in the first year [10], better medical degrees [5] and possibly post-graduate career progression.[11] Most medical schools therefore require a minimum of two A grades in science, and one B grade, or equivalent tariff points, in another subject.[4] Many students offer a third science subject, some a fourth.

Competition for admission to medical school in the UK is intense and selection is becoming increasingly difficult.[12] At Nottingham, the selection procedure includes academic and non-academic criteria which have been shown to predict success on the medical course. [5-7] In 2000, we required applicants for the 5-year medical course to have an A grade in Chemistry and another science, and at least a B in any additional subject (excluding general studies). Non-academic criteria (comprising personal statements and references from the UCAS form, an online questionnaire of extracurricular activities, and interview performance) were scored.

Cowley has recently proposed that students who study a non-science subject would be better prepared for the medical course, and such subjects should be compulsory.[13] He suggests that studying English Literature in particular would develop students' capacity to understand individual patients, to communicate skilfully, and to be better able to discuss complex ethical issues. However, these arguments are made without demonstrable evidence from medical school performance.

### The context of this study – the University of Nottingham 5-year undergraduate course

The course comprises pre-clinical and clinical components. Topics for the first two years (Part I, pre-clinical) are grouped into four Themes: A (The Cell), B (The Person), C (The Community), and D (The Doctor). Of these, A and B are largely biochemical and physiological science-based whilst C and D are behavioural, social and clinical science-based. A wide variety of techniques are used to assess students' progress. Themes A and B are assessed extensively over the 2 years; examinations consist predominantly of blocks of true/false questions, short answers, short essays, and single word (or phrase) answers, with some course assessments. These can all be grouped as 'knowledge-based', and averaged as a proxy for 'pre-clinical knowledge'.

Theme C (Behavioural Sciences, Public Health and Disease Epidemiology, and 'Services, Clients & Community'), is assessed four times, with short answer and true/false questions, plus a prepared essay of 2000 words. Theme D (Communication Skills, and Early Clinical & Professional Behaviour) is assessed each semester by in-course appraisal. In addition there is an OSCE-format assessment of communication and early clinical skills in Semesters 2 and 4, and presentation skills assessment in semester 4. (OSCE = Objective Structured Clinical Examination; skills are assessed by role-play in various standardised situations). Although Theme C is examined by means of written assessments, the subject matter is less scientific than in Themes A and B, and therefore we have grouped the results with those of Theme D as a proxy for 'pre-clinical skills'.

Pre-clinical study in the first half of the third year (Part II) consists of a research project, including dissertation and viva, and several taught courses, all of which contribute to the marks for the award of BMedSci (Bachelor of Medical Science) at the end of the third year.

Clinical study is in three phases. Phase 1 (second half of the 3rd year) concentrates on general adult medicine and surgery. Phase 2 (4^th ^year) contains specialties such as child health, obstetrics and gynaecology and psychiatry. Phase 3 (5^th ^year) encompasses advanced medicine, surgery, musculo-developmental disorders and disability, and general practice. All these attachments have formal written examinations, which generally consist of objectively-marked questions (OMQs) of various types, and constitute a score for 'clinical knowledge'. In addition there are practical examinations in the form of OSCEs and/or OSLERs (Objective Structured Long Case Examination Record) to assess clinical and communication skills for the medical and surgical attachments, child health, obstetrics & gynaecology, and psychiatry. These generate a score for 'clinical skills'.

Students on the course are therefore subject to a wide variety of assessment types, but they can be grouped broadly into knowledge and skills in both the pre-clinical and clinical course. Part II (the research project) includes quite specific additional skills and therefore we have considered this separately.

As part of our ongoing evaluation of admission and selection procedures, we therefore investigated whether the choice of subjects at A-level influenced performance on the University of Nottingham medical course, for one entry-year cohort of students who progressed normally to graduation.

## Methods

### Subjects

The study concerned one year-group of students who were admitted to the 5-year undergraduate medical course at Nottingham University and progressed normally to graduation.

### Data collection and preparation

Routinely-collected data were used for this study. Basic pre-admission socio-demographic information (age at course entry, sex, ethnic group and fee status) were collated from the Medical School and central University databases. Information on the subjects and grades passed at A-level were extracted from the students' UCAS forms. Assessment marks throughout the course were added to these data, and then personal identifiers were replaced by a unique study number.

Data were coded as required to create binary variables and avoid any statistical problems with non-normal distributions, such as those of age and average tariff score. Choice of A-level subjects was grouped as all-science (any combination of biology, chemistry, physics and maths), or non-science (inclusion of any other subject, such as a language or humanity). A-level tariff points for each subject (A = 120, B = 100 etc) were used to calculate the average tariff point score. A higher/lower tariff point indicator was created, using <110 points as the cut-off for 'low score'. (All students with the grades of AAB or above would score an average of at least 110 points). Ethnic groups were collapsed into White or non-White. Age was grouped as younger (19 or below) or older (over 19), and fee status (reflecting domicile) as home/EU or overseas.

### Course outcome variables

We used average percentage marks for the following groups of assessments to create five course outcomes;

1. Themes A&B (pre-clinical knowledge) (years 1 & 2)

2. Themes C&D (pre-clinical skills) (years 1 & 2)

3. Part II (research and writing skills, and knowledge) (year 3)

4. Clinical knowledge (based on MCQ assessment in years 3–5)

5. Clinical skills (OSCEs and/or OSLERs in years 3–5)

These were all normally distributed.

We examined whether performance varied between students with and without a non-science A-level, and with three or four A-levels. Since tariff point scores and socio-demographic variables are known to affect performance, these were included as explanatory variables. We also examined whether performance in the pre-clinical course or Part II might predict subsequent performance.

### Statistical analysis

Data analyses were carried out in SPSS v15. Binary predictor (explanatory) variables were sex, ethnicity, fee status, age group, taking three A-levels or four, taking a non-science or not, and the tariff point indicator. The outcome (dependent) variables were the course outcomes listed above.

Univariate comparison of the binary predictor variables against all course outcomes was conducted with t-tests. Given the number of comparisons, the Bonferroni correction for multiple tests was applied (p considered to be significant only at = < 0.001).

Multivariate analysis for independent predictors of course outcome was conducted using hierarchical multiple linear regression. Predictors were entered in three stages, in this order:

1. socio-demographic variables

2. A-level variables

3. progressive course outcomes, ie Themes A&B and C&D as predictors for Part II, and Themes A&B, C&D, and Part II as predictors for the Clinical course.

A number of students did not graduate as expected within five years and were excluded from the analyses. Their pre-admission characteristics were compared with the study cohort using the Chi-square test (binary predictor variables). As confirmation, we used a binary logistic regression model.

### Ethical approval

This analysis of aggregated, anonymised routinely-collected data was approved by the Chairman of the University of Nottingham Research Ethics Committee without formal submission.

## Results

### General

A total of 210 students entered the 5-year Nottingham medical course in October 2000. Of these, 46 were excluded from the main study for the following reasons:

• 39 students who did not progress normally on the course. This group comprised 25 graduating late after suffering academic or health problems, 12 voluntary withdrawals, and two whose course was terminated after academic failure in Year 1

• seven students who had unknown A-level subjects, had fewer than two science subjects, or had overseas qualifications

Table 1 describes the study cohort. There were no statistically significant differences between the study cohort and excluded students for any of the binary variables shown (Chi-square tests).

**Table 1:** The entry cohort and study/excluded groups

	**Pre-admission characteristic**	**entry cohort** **n = 210**		**study cohort** **n = 164**		**excluded students** **n = 46**	
		**n**	**%**	**n**	**%**	**n**	**%**

Gender	Male	90	43	71	43	19	41

	Female	120	57	93	57	27	59

							

Ethnicity	White	149	71	117	71	32	70

	Non-White	53	25	42	25	11	24

	Not declared	8	4	5	3	3	6

							

Age group	19 or under (Younger)	184	88	147	90	37	80

	Over 19 (Older)	26	12	17	10	9	20

							

Fee status	Home or EU	182	87	143	87	39	85

	Overseas	28	13	21	13	7	15

							

A-level subjects	Non-science A-level	61	29	45	27	16	35

	All science A-levels	147	70	119	73	28	61

	Other/unknown	2	1			2	4

							

Number of A-levels	4 A-levels	82	39	61	37	21	46

	3 A-levels	126	60	103	623	23	50

	Other/unknown	2	1			2	4

							

Average tariff point score	Higher (> = 110)	158	75	128	78	30	65

	Lower (<110)	42	20	33	20	9	20

	Unknown	10	5	3	2	7	15

Specifically, there were no significant differences in the A-level profiles between the two groups. Although the raw score for tariff point average was slightly lower in the excluded students (median 113 vs 120, Z = -3.09, p 0.002, Mann Whitney U test), the proportions with a lower score were not different.

When these variables were entered into a binary logistic regression model, there were no independent predictors of being an excluded student.

### Univariate analyses

Table 2 illustrates the mean marks obtained throughout the course in relation to the pre-admission variables. Whilst female, White and Home/EU students tended to perform slightly better than male, non-White, and Overseas students, the only statistically significant differences after the Bonferroni correction were for students with higher tariff point averages. They performed better in most parts of the course, particularly in the knowledge-based assessments, but not in clinical skills. Importantly, there were no differences in performance between students with or without a non-science A-level or with three or four A-levels.

**Table 2:** Pre-admission characteristics and mean course marks

**Pre-admission characteristics**		**Themes A&B average**		**Themes C&D average**		**Part II average**		**Clinical Knowledge average**		**Clinical Skills average**	
		**Mean**	**SD**	**Mean**	**SD**	**Mean**	**SD**	**Mean**	**SD**	**Mean**	**SD**

Gender	Male	61.9	6.9	63.9	5.2	65.5	4.9	59.6	5.5	65.2	6.4

	Female	61.5	6.1	66.2**	4.8	66.0	4.3	60.8	5.3	67.6*	6.3

											

Ethnicity	White	61.2	6.6	65.8*	4.6	65.8	4.6	60.4	5.6	67.2*	6.6

	Non-White	62.9	6.1	63.8	5.9	65.7	4.4	59.9	5.0	64.9	5.8

											

Age group	19 or under (Younger)	61.3	6.2	65.3	5.1	65.7	4.5	60.3	5.5	66.6	6.6

	Over 19 (Older)	65.2 **	7.5	64.5	5.2	66.8	4.5	60.4	4.6	65.9	5.6

											

Fee status	Home/EU	61.5	6.7	65.6*	5.1	65.9	4.6	60.6*	5.6	67.2**	6.3

	Overseas	62.6	4.3	62.5	4.6	65.1	4.1	58.3	3.7	62.4	6.2

											

Non-Science A level	Non-science A level	61.9	7.3	65.7	4.5	66.8	4.3	61.4	5.5	68.0	5.9

	All science A levels	61.6	6.1	65.0	5.3	65.4	4.6	59.9	5.3	66.0	6.6

											

Number of A levels	4 A levels	62.2	5.8	65.5	5.4	66.1	4.8	61.3	4.5	67.1	6.8

	3 A levels	61.3	6.8	65.0	4.9	65.6	4.4	59.7	5.8	66.2	6.3

											

Average tariff point score	Higher (110 or above)	63.1***	5.9	65.9**	5.1	66.2*	4.4	61.1***	5.2	66.9	6.7

	Lower (<110)	56.3	5.7	63.1	4.2	64.1	4.5	57.1	5.0	65.5	5.8

Key: t-tests, * p = <0.05; ** p = <0.01; *** p = <0.001 before Bonferroni correction

t-test statistics are shown after applying Levene's test for unequal variance, if appropriate

### Multivariate analysis

Table 3 shows the significant independent predictors of course performance, determined by hierarchical multiple linear regression. A statistically significant change in R^2 ^(ΔR^2^) indicates that the additional block of variables adds significantly (in terms of variance) to the prediction of the outcome model.

**Table 3:** Significant independent predictors of course performance (hierarchical multivariate linear regression analysis)

**Outcome variable**	**Predictor variable block^†^**	**R^2^**	**ΔR^2^^‡^**	**Significant predictors^§^**	**Beta**	**t**
Themes A&B	1 (socio-demographic)	0.063	0.063*	Younger age group	-0.237	-2.92 **

	2 (A-levels)	0.263	0.200***	Younger age group	-0.262	-3.57 ***

				Higher tariff average	0.443	6.28 ***

						

Themes C&D	1 (socio-demographic)	0.104	0.104**	Home/EU fee status	0.183	1.98 *

				Female gender	0.225	2.95 **

	2 (A-levels)	0.171	0.068 **	Home/EU fee status	0.248	2.70 **

				Female gender	0.231	3.11 **

				Higher tariff average	0.246	3.29 **

						

Part II	1 (socio-demographic)	0.015	0.015	(none)		

	2 (A-levels)	0.078	0.063 *	Higher tariff average	0.205	2.59 *

	3 (Themes A&B and C&D)	0.244	0.165 ***	Higher ave Themes C&D	0.390	4.30 ***

						

Clinical knowledge	1 (socio-demographic)	0.039	0.039	(none)		

	2 (A-levels)	0.178	0.139 ***	Home/EU fee status	0.279	3.04 **

				Higher tariff average	0.325	4.36 ***

	3 (Themes A&B and C&D, and Part II)	0.479	0.301 ***	Higher ave Themes A&B	0.503	6.27 ***

				Higher ave Part II	0.144	2.13 *

						

Clinical Skills	1 (socio-demographic)	0.315	0.076 **	Home/EU fee status	0.239	2.59 *

				Female gender	0.199	2.61 *

	2 (A-levels)	0.353	0.084	Home/EU fee status	0.269	2.84 **

				Female gender	0.197	2.59 *

	3 (Themes A&B and C&D, and Part II)	0.561	0.269 ***	Higher ave Themes C&D	0.317	3.45 **

^† ^denotes the successive addition of variable blocks to the hierarchical regression, as defined in the Methods

^‡ ^a significant value in this column it indicates that the additional block of variables adds significantly (in terms of variance) to the prediction of the outcome variable.

^§ ^denotes the significant predictors found as each block of variables was added to the model

* p < 0.05, ** p < 0.01, *** p < 0.001

There was no evidence that having a non-science A-level affected performance at any stage on the course, nor that the number of A-levels was influential.

In the pre-clinical course, a higher A-level tariff point average was independently predictive of a better performance in both Themes A&B (together with older age) and Themes C&D (together with female gender and Home/EU fee status).

In Part II, there were no socio-economic predictors. Tariff point score was predictive after Block 2 variables were added but this was overridden by pre-clinical skills performance (Themes C&D) in the final model.

The prevailing influence of prior course performance was shown even more clearly in the clinical course. Predictive effects of socio-demographic and A-level variables were observed initially but lost when block 3 was added, with Themes A&B and C&D predicting clinical knowledge and skills, respectively.

Overall these analyses indicate that demographics and A-levels may influence early performance but become less important as the course proceeds.

## Discussion

This study of a single medical undergraduate cohort showed that studying a non-science A-level subject did not influence course performance at Nottingham University. Studying four subjects rather than three was not influential, although a higher tariff point average appeared beneficial. When earlier course performance was included in the analysis, this became the most consistent positive predictor of subsequent assessments. These results support our admissions policy, which includes academic standards but does not specify or discriminate between the choice of third or fourth A-level subject.

### Limitations of the study

This study was performed on a single cohort of students at a single University, studying an integrated, systems-based course. We were constrained by the time-frame of the work, which was a BMedSci research project so necessarily small in scale. It was therefore not practicable to increase the sample size by studying several consecutive cohorts.

The study group represented only 164 of 210 (78%) who began the course. The majority of the excluded students (39) had failed to make normal progression on the course, and we also had to exclude seven for whom we had no valid A-level scores. Although the excluded students were comparable in socio-demographic terms, their average tariff point scores were lower. This was not statistically significant, but numbers are small. The subsequent performance at medical school of the 39 who were excluded from the study for academic failure might have influenced the results had they been included. However, the validity of the analyses would then have been adversely affected in two ways: the numbers (denominators) would have decreased over time as students left the course; and those dropping back would have taken similar but different examinations in subsequent academic years.

Our results therefore may not necessarily be fully generalisable to other years at Nottingham, or to other Universities with different admissions criteria and different courses, but they do reflect the study of a homogeneous group of successful students.

The relatively small numbers and the resultant grouping of A-level qualifications meant that we could not identify the effect of studying individual subjects, nor the effects of a non-science A-level as a third or as a fourth subject. Similarly, the condensation of ethnicity into White or non-White precludes comment about different ethnic subgroups. Although we are confident that our analyses are valid within their context, is possible that these factors might be shown to be important in a substantially larger study.

The final sample of 164 participants might be considered small to identify any effects of A-levels. However, other well established small effects (eg demographic) were observed in this cohort, supporting its overall validity.

### A-level subjects and performance

In 2000, Chemistry was the only stipulated A-level at Nottingham. Subsequently Biology was added as the second compulsory subject because we found a positive correlation of Biology grade and course performance.[5] We are only aware of one other study addressing the influence of A-level subject choice, which reported no influence of 'all science' or 'mixed' subjects on performance in a 3^rd ^year skills examination.[14] This is in agreement with our results.

We can see no *a priori *reason why subject choice beyond chemistry and biology should be particularly influential, since succeeding on the course and beyond requires many attributes in addition to academic ability, such as personal and motivational qualities. [15,16] A student's choice of A-level subjects may be influenced by many factors, including school policy and optimising chances for medicine. The potential effects of studying a non-science A-level could be two-fold: an increase in empathetic and communication skills; and better abilities with tasks that involve essay-writing and discussions as opposed to the recall of factual knowledge. If this were the case, then we would have expected to see some dichotomy between the performance of students with and without a non-science A-level in those parts of the course that assess these qualities, ie pre-clinical and clinical skills. No such evidence was found. Cowley also suggests that students who are unwilling to study a non-science subject may be better suited to a research career than medicine.[13] This seems to be an unsubstantiated assumption and there was no relationship in our study between A-level choice and BMedSci performance, part of which involves a research project. There is an increasing need to attract and retain doctors into academic medicine and the approach should be all-encompassing rather than exclusive. [17]

A higher A-level grade point average has been found elsewhere to predict better performance in medical degrees [5,12,15] and in higher education overall.[18] We are reassured by our finding that the actual number of subjects studied is not important; we would not wish to advantage or disadvantage students who took either three or four subjects since some may be encouraged or even constrained by an individual school's policy.

In 2000, post-16 education was changed, such that students study a wider combination of subjects at AS (Advanced Subsidiary) level for one year, before focusing on three or four at A-level (termed A2) in the second year.[19] Many current medical students will therefore have studied at least one non-science subject. However, AS qualifications are not considered during the admission process at Nottingham. Students who are obliged to study a non-science at AS, but would otherwise not choose one, may not fully engage with it, so such study may not be an accurate reflection of their interests or capabilities.

### The effects of socio-demographic variables

This study confirms previous work by ourselves and others, showing that females tend to perform slightly better on the course [8,9], and particularly within skills assessments.

[20,21]This may relate to differences in personal attributes between men and women.[22-24]

Previous studies at Nottingham have shown that minority ethnicity is a predictor of 'struggling' and poor performance [8,9], whereas fee status is not; in this self-selected cohort, in which the strugglers and worst-performing students had been excluded from the analysis, ethnicity was no longer a predictive factor, although Home/EU students were more likely to perform well, particularly in the clinical course. This may reflect a subtle balance between ethnicity and fee status, and also underlying cultural and language effects, as found in numerous other studies. [25,26]

### The effects of prior course performance

Our results confirm and extend data from our previous work.[6] By using separate averages for Themes A&B and C&D, representing pre-clinical knowledge and skills, respectively and also for clinical knowledge and skills, we have demonstrated a predictive link between students' strengths in knowledge or in skills. Hierarchical multiple linear regressions showed that prior performance replaces the pre-admission predictors and increases the predictive power of the model for the clinical years. Part II, the BMedSci, requires a variety of different attributes and has been shown before to be less well related to conventional factors.[9] Its association with pre-clinical skills assessments may perhaps reflect better writing skills.

### Suggestions for future research

The crucial question is whether studying a non-science subject will create a better doctor, as Cowley has suggested. That is difficult or impossible to answer since 'good' doctors develop over the span of their careers, not just within their 5 years at medical school. However, in the interests of furthering the debate on admission and selection processes, we would suggest further studies on larger cohorts, to investigate in more depth any potential influence of specific non-sciences such as languages. It would also be interesting to carry out this study within newer types of course (eg problem-based learning, PBL), and on Graduate Entry students who have very different backgrounds.

## Conclusion

This study provides no evidence that our current admissions policy regarding A-level subjects should be changed. A student's additional qualifications, over and above Biology and Chemistry, do not convey any advantage or disadvantage in terms of course progress.

## Competing interests

The authors declare that they have no competing interests.

## Authors' contributions

All authors contributed to the planning and development of the project, the interpretation of the results, and the writing of the paper. DJ led the study and supervised JS, who undertook the analysis for her BMedSci project. JY prepared the anonymised datafile and undertook additional analyses. EF provided statistical advice. All authors read and approved the final manuscript.

## Pre-publication history

The pre-publication history for this paper can be accessed here:


